# Advances in Polymer Based Composite Coatings

**DOI:** 10.3390/polym13101611

**Published:** 2021-05-17

**Authors:** Andreea Groza

**Affiliations:** National Institute for Laser, Plasma and Radiation Physics, 409 Atomistilor Street, P.O. Box MG36, Magurele, 077125 Bucharest, Romania; andreea.groza@inflpr.ro

Polymer based composite coatings represent an important class of materials for various applications [1,2,3,4,5,6,7,8,9] due to their capabilities to couple the elastic, optical, or wettability properties of the polymers with the mechanical, physical, and chemical properties of metals, dielectrics, or ceramics. Usually, selective chemical or plasma methods are chosen for synthesis or tailoring of the surface properties of polymer based layers. The authors who submitted their latest results to this Special Issue [1,2,3,4,5,6,7,8,9] evidenced the advantages as well as the drawbacks of the following methods used for different coating synthesis procedures: atom transfer radical polymerization technique [1], electrospraying [2], radio frequency magnetron sputtering technique [3,8] chemical vapor deposition [4,6], plasma enhanced chemical vapor deposition [4], and high-pressure spray gun [7]. There is also one particular study on the improvement, experimentally and theoretically, of the blister technique used for measuring of the polymers’ adhesion strength [5].

Summarizing, this Special Issue contains eight papers dedicated to synthesis and analysis of mechanical, wettability and physicochemical properties of different kind of polymer based composite coatings [1,2,3,4,5,6,7,8]. It also comprise one review paper which provides an overall insight to all the class of polymer coatings with applications in the marine industry [9].

By atom transfer radical polymerization technique (ATRP), Guazzelli E., et al. [1], synthesized amphiphilic diblock copolymers, consisting of two blocks of polystyrene modified with a mixed PEG-fluoroalkyl side chain formed by ~12 CH_2_CH_2_O and ~4 CF_2_CF_2_ groups, as additives for elastomeric SEBS two-layer films. They are biocide free, with low bulk elastic modulus and low energy being of high interest in the fight against marine biofouling [9]. The hydrophobic character of the samples immersed in water was attributed to the fluorine enriched film surfaces. By the length of the PEG segment and degree of polymerization of the second polymer block, the authors controlled the contraphilic response of the diblock copolymer in contact with water.

The study of Venkataraman M., et al. [2], showed the importance of the water content in a polytetrafluoroethylene (PTFE) particles and carbon microparticle solution prepared for generation of microporous layers by electrospraying technique. Even if the carbon particles do not influence the surface and volume resistivity of the layers, their presence in the solution increases the degree of ionization, providing a better shape control of the material during the electrospraying process. The size distribution of the PTFE layers on the surface of the substrate was explained by connecting the roughness of the layers with their hydrophobicity. Thus, the highest values for the layer roughness and hydrophobicity were obtained for a percentage of 45% water content in the PTFE-carbon particle solution.

Satulu V., et al. [3], reports on the synthesis of polymer nanocomposite by radio frequency magnetron sputtering technique, providing proofs for the use of PTFE/Ag/PTFE coatings as antireflective layers in technological applications, such as biosensors or optoelectronic devices [3]. The top-covered PTFE layer acts as a barrier against oxidation of Ag spherical nanoparticles, conducive to an enhancement in the intensity of the 430 nm surface plasmon resonance peak as its thickness increases.

Yang R., et al. [4], showed that wood samples functionalized with polydimethylsiloxane by low temperature chemical vapor deposition (CVD) technique can be produced. Dichlorodimethylsilane gas and water vapor was used for injection of polydimethylsiloxane (PDMS) into poplar wood samples. A chemical pretreatment of wood samples for the weakening of the cellulose, hemicellulose, and lignin bonds has been made, in order to stimulate the penetration of dichlorodimethylsilane vapors into the wood cells and walls. The PDMS coating deposited on the wood samples increased it thermal stability by reducing the wood mass weight loss at low temperatures. Moreover, the authors obtained PDMS@wood samples with hydrophobic properties stable in time and excellent mechanical characteristics. The best water repellent properties of the surface of the PDMS@wood were obtained after 1 h at 70 °C treatment at a contact angle of 156.0°.

Yong-Sheng Lian et al. [5], developed, in their paper, an improved theory for the determination with high accuracy of energy release rate formula (energy released per unit delamination area) and a new experimental set-up for pressurized blister technique of elastic layers deposited on rigid substrates. The delamination area of the coating from substrate by blister test technique has the benefit of an axisymmetric geometry of the cracks caused by the uniformity of the applied pressure. The advantage of the proposed experimental set-up is that the two vessels used for pressurized blister tests in which the liquids are poured have the same radius and a valve between the connecting pipes. Thus, the pressure fluctuations due to the liquid pouring during measurements is avoided [5]. Consecutively, the accuracy of the measurements and calculation energy release rate is improved. The validity of the new theory for the measurement and calculation of the adhesion strength of elastic coatings on rigid substrates was tested in comparison with the previous existent ones [5].

The most known feature of the plasma enhanced CVD (PECVD) technique is that the generated polymeric films are chemically stable and have strongly cross-linked structures, in comparison with the polymers produced by initiated chemical vapor deposition method (iCVD) which are linear. However, the retention of chemical functional groups is higher for materials treated by iCVD than PECVD [6], as the degree of polymerization counts a lot. In this context, Zhi Chien Ng et al. [6], in their study, analyzed in detail the physicochemical and hydrophobic properties, as well as oil/solvent absorption capabilities of polyurethane (PU) foams, polyurethane foams functionalized with perfluorodecyl acrylate (PFDA), 2,2,3,4,4,4-hexafluorobutyl acrylate (HFBA), and hexamethyldisiloxane (HMDSO), by iCVD and PECVD techniques. Basically, the authors investigated the influence of F and Si content on the hydrophobicity of functionalized PU foams produced by the two methods. The long fluorine branched polymers had higher contact angle values. The authors showed that, for a deposition time of 5 min, the absorption capabilities of PU foams functionalized with PHFBA by iCVD to chloroform, acetone, cyclohexane, and edible oil are higher than those produced by PECVD. The authors conclude that the reusability of PU/PHFBA foams (produced by iCVD) to absorption of chloroform, acetone, cyclohexane, and edible oil is more proper for practical applications than that of unmodified PU foams.

Jiaxuan Niu et al. [7], synthesized fluorocarbon/silica composite layers with superhydrophobic properties for scavenging energy in sliding mode from raindrops. A static contact angle of 156.2°, as well as a sliding angle of 6.74° for the coatings, was measured. The adherence of the coatings has been improved by mixing the nanosilica with fluorocarbon emulsion prior to spraying with the high-pressure spray gun. Thus, the adhesion grade has been increased from 0 to 1 (according to the adhesion grade standard of paints and varnishes-cross cut test for films—GB/T9286-88) when nanosilica was added to the fluorocarbon layers. The most interesting result of the work seems to be the triboelectric energy harvesting capabilities of the fluorocarbon/silica composite superhydrophobic coating. By attaching an aluminum electrode to a fluorocarbon/silica composite layer that cover an ITO substrate, an increase in the voltage of the open circuit from 0.2 V to 20 V for 4.5 A short circuit current was obtained. Thus, one water drop sliding on the fluorocarbon/silica composite coating generates enough electrical power to light up 16 commercial LEDs.

Groza A. et al. [8], by using a radio frequency magnetron source for sputtering of a hydroxyapatite-chitosan (HApCs) composite target, obtained HApCs layers with grain-like structures of spherical shapes and microns to hundreds of nm sizes, as a function of Ar gas working pressure. The morphology of the surface of HApCs layers was explained on the assumption that the coagulation of HApCs macromolecules on the substrate takes place as the temperature measured at the substrate surface is about 523 K in comparison with the plasma temperature of ~20,000 K. Because the nucleation and coagulation stages of polymer nanoparticles in plasma are processes governed by negative charges, their densities at the substrate [8] also influence the polymer nanoparticle shapes and sizes. It was evidenced that the nm sizes of the grain like structures detected on the surface of the HApCs layers increase with the electron number density of the deposition plasma.

The review of Pistone A et al. [9], draws attention to the importance of superhydrophobic coatings and their mechanical properties in the maritime industry. The accumulation of microorganisms, barnacles, and seaweeds on the ship hulls hampers the proper hydrodynamic movement of the boats. Therefore, by covering the external surfaces of boats with proper composite coatings, the deposition of marine organisms could be avoided. They also point out that the anticorrosive and free of biocide (mainly copper and Zn metals) coatings can protect the marine environments. These types of superhydrophobic coatings contain mainly epoxy and siloxane resin, epoxy modified poly-siloxane based resins, or polyurethane. The development of such coating by different physical and chemical methods can represent viable “green” solutions for replacement of the paints based on heavy metals or toxic biocides currently used for antifouling and biofouling of the surface of submerged structures of ships.

At the end of this Editorial, I would like to address my appreciations to all authors who contribute with their most new research studies to this Special Issue. I would like to also thank the Polymers Editors who invited me to conduct a Special Issue in Polymers Journal as a Guest Editor. I acknowledge all the reviewers for their efforts, proper comments, and critical point of views. Finally, I thank all the assistant editors, especially Ailes Gao who assisted and supported me during the entire editing process.
